# A Review of the Relationship between the Immune Response, Inflammation, Oxidative Stress, and the Pathogenesis of Sickle Cell Anaemia

**DOI:** 10.3390/biomedicines11092413

**Published:** 2023-08-29

**Authors:** Florence Ifechukwude Aboderin, Taofeeq Oduola, Glenda Mary Davison, Oluwafemi Omoniyi Oguntibeju

**Affiliations:** 1 Department of Biomedical Sciences, Faculty of Health and Wellness Sciences, Cape Peninsula University of Technology, Bellville 7535, South Africa; udoifechukwude@oauife.edu.ng; 2 Department of Chemical Pathology, Usmanu Danfodiyo University, Sokoto 840004, Nigeria; oduola.taofeeq@udusok.edu.ng; 3 SAMRC/CPUT Cardiometabolic Health Research Unit, Department of Biomedical Sciences, Faculty of Health and Wellness Sciences, Cape Peninsula University of Technology, Bellville 7535, South Africa; davisong@cput.ac.za

**Keywords:** sickle cell anaemia, chronic inflammation, immune system, oxidative stress, haemolysis, blood transfusion

## Abstract

Sickle cell anaemia (SCD) is a life-threatening haematological disorder which is predominant in sub-Saharan Africa and is triggered by a genetic mutation of the β-chain haemoglobin gene resulting in the substitution of glutamic acid with valine. This mutation leads to the production of an abnormal haemoglobin molecule called haemoglobin S (HbS). When deoxygenated, haemoglobin S (HbS) polymerises and results in a sickle-shaped red blood cell which is rigid and has a significantly shortened life span. Various reports have shown a strong link between oxidative stress, inflammation, the immune response, and the pathogenesis of sickle cell disease. The consequence of these processes leads to the development of vasculopathy (disease of the blood vessels) and several other complications. The role of the immune system, particularly the innate immune system, in the pathogenesis of SCD has become increasingly clear in recent years of research; however, little is known about the roles of the adaptive immune system in this disease. This review examines the interaction between the immune system, inflammation, oxidative stress, blood transfusion, and their effects on the pathogenesis of sickle cell anaemia.

## 1. Introduction

SCD is a global health issue affecting millions of people [1,2]. The highest prevalence is in Africa with Nigeria being identified as the epicentre, having 4 to 6 million affected individuals [3]. Fraiwan et al. [4] reported that approximately 150,000 children with SCD are born each year in Nigeria with 70–90% dying before the age of five.

Sickle cell disease (SCD) is a genetic haemoglobinopathy which is passed down to children from parents who are carriers [5]. Haemoglobin SS is the most clinically severe subtype [6,7,8], and it is well established that the disorder is not only a rheological disease but is also characterised by chronic inflammation and oxidative stress. These processes lead to the development of vasculopathy and several other chronic complications [9]. SCD is caused by a single-point mutation in the gene encoding for the β-globin chain resulting in the replacement of glutamic acid with valine [10,11]. The consequence of this mutation is the production of an abnormal haemoglobin (HbS), which polymerises in low oxygen concentrations [12,13] and results in the dysfunction and deformation of the red blood cells to a sickle shape form [2,9].

Haemoglobin polymerisation triggers a sequence of events leading to several complications, including vascular–endothelial dysfunction, anti-inflammatory (nitric oxide) deficiency, inflammation, oxidative stress, hypercoagulability, and recurrent immune cell activation [2,14,15]. These events result in an elevation of free radicals through the release of free haemoglobin, heme, and activation of pro-oxidant enzymes [2,16]. Excessive free radicals contribute to increased oxidative stress, which induces chronic inflammation and a reduced life expectancy [17]. 

Patients with SCD require regular blood transfusions, which increase exposure to foreign antigens and elevate the risk of producing alloantibodies that may cause delayed haemolytic transfusion responses and make it difficult to find suitable blood [18,19,20]. Moreover, it has been reported that multiple blood transfusions may lead to transfusion-transmitted infections, inflammation, and iron overload [21,22]. These factors all influence the pathogenesis and development of SCD; however, the consequences of a dysregulated immune system are complex, and therefore, further investigation is required [23,24]. 

The innate and adaptive immune responses in SCD are impaired and cannot effectively protect against infection [25,26] with some researchers reporting that the persistent activation of the innate immune system results in the generation of excessive amounts of reactive oxygen species (ROS) (13, 28–30). According to Ahmad and Ahsan [27], Engwa et al. [28] and Atiku et al. [10] elevated amounts of reactive species induce oxidative stress and tissue injury, while other reports have revealed that the adaptive immune cells are also dysfunctional, resulting in lower antibody levels compared to healthy individuals [24]. In addition, preliminary studies have revealed that the number of T and B cells, as well as their function, are impaired [24,26,29]. Based on these investigations, this review aims to examine the current literature and provide an updated understanding of the relationship between the immune response, inflammation, oxidative stress, blood transfusion, and the pathogenesis of SCD.

## 2. Immune Mechanisms Involved in the Pathogenesis of Sickle Cell Anaemia 

Leukocytes such as neutrophils, eosinophils, basophils, monocytes, lymphocytes, and platelets have been implicated in the pathogenesis of SCD, as evidenced by several studies [23,24,30,31]. These cells are reported to be responsible for promoting inflammation, adhesion, and the painful crises characteristic of SCD [23,32]. Even in the absence of infection, leukocytosis and immune activation is a common phenomenon. In support of this, studies using flow cytometry were adopted to analyse peripheral blood neutrophils for the expression of CD18. CD18 is upregulated during inflammation and binds to the adhesion molecules ICAM-1 and ICAM-4 on the endothelium, resulting in activation and inflammation. These experiments revealed that CD18 expression was increased in SCD patients and that the neutrophils had a higher affinity for the vascular endothelium and increased adherence, which resulted in the recruitment of sickled red cells and an elevated risk of vaso-occlusive crises (VOC) [33,34]. 

Further contributions have demonstrated that polymorphonuclear leukocytes (PMNs) have high CD64 expression and elevated levels of L-selectin, SCD 16 and elastase, resulting in further amplification of the adhesiveness to the endothelium [35]. According to Antwi-boasiako et al. [36], both male and female SCD patients who experience complications have noticeably higher leukocyte counts than their healthy counterparts with the white blood cell count frequently being used by clinicians to predict stroke and acute chest syndrome [37]. Free haemoglobin and heme released during haemolysis have been identified as key players in the activation of the innate and adaptive immune response [9,23] with reports suggesting that patients with high haemolysis rates are at greater risk of early mortality [2,19]. The continual breakdown and destruction of red blood cells result in sustained activation of innate immune cells resulting in a chronic inflammatory state [24,38,39].

Endothelial cells are one of the first cell types to be activated in the presence of heme. Heme activates endothelial cells inducing the expression of adhesion molecules (E-selectin, intercellular P-selectin, vascular cell adhesion molecule 1) which initiates the activation and recruitment of other immune cells, including macrophages, neutrophils, mast cells, and platelets. The activated macrophages secrete several pro-inflammatory cytokines, including IL-1β, through stimulation of the NLRP3 inflammasome which further contributes to the creation of a pro-inflammatory and pro-coagulant environment [23]. Consequently, this sustained inflammatory state results in a VOC which is commonly described in patients with SCD [15,40].

Heme also has a direct link with the activation of neutrophils by acting as a prototypical pro-inflammatory molecule and recruiting neutrophils to the site of injury via the stimulation of protein kinase C and ROS generation [2,15]. In addition, heme inhibits neutrophil apoptosis via the modulation of phosphoinositide 3-kinase and NF-κB signalling, which further contributes to the development of chronic inflammation [41]. Neutrophils have been identified as playing a significant role in the development of VOC with increased counts being associated with clinical complications, including earlier death and haemorrhagic stroke [40].

Platelets, which are small anucleate cells and play a role in the immune response, have also been implicated in the pathogenesis of SCD. Malik [42] and Nolfi et al. [43] reported that platelet activation together with a decrease in nitric oxide (NO) is triggered by the release of heme into the circulation. Once activated, the platelets release several soluble mediators such as CD40 ligand and thrombospondin which have the potential to initiate thrombosis and pulmonary hypertension [23,42]. Molecules secreted by the platelet bind to CD36, also known as glycoprotein IV, on sickled RBCs and endothelial cells. Platelets go on to associate and bind to other immune cells, including neutrophils, macrophages, and monocytes [39]. It has been demonstrated that activated platelets bind to neutrophils and monocytes in a P-selectin signalling pathway to form aggregates that promote VOC, inflammation, and thrombosis through various mechanisms [30]. In SCD mice, Allali et al. [23] reported that platelet–neutrophil aggregates may be an important factor in the development of pulmonary arteriole micro-emboli.

Although many studies have investigated the role of the innate immune system, the role of the adaptive immune response is still poorly understood. Studies performed on human and animal subjects have reported that in SCD, both T and B lymphocytes are dysfunctional [44,45]. To further analyse this, the relationship between splenic size and lymphocyte counts has been investigated by several researchers [24,46,47,48,49]. Ojo et al. [50] used flow cytometry to analyse CD4+ T-lymphocytes in blood samples from 40 steady-state SCD patients and correlated the counts with ultrasonography used to determine spleen size. They reported that in patients with auto-splenectomy, the mean CD4+ count was not significantly different to HbS patients with a normal-sized spleen. Several studies have further investigated the effect of hydroxyurea (HU) on lymphocyte subset counts [45,51,52] and have shown that SCD patients receiving HU had lower total lymphocytes, T cells, CD4+ T cells, memory CD4+ T cells, and memory CD8+ T cells compared to those who were untreated [24]. These findings may be explained by the fact that HU is often used as a chemotherapeutic drug and acts by inhibiting DNA synthesis, resulting in cytotoxicity and cell death.

Alloimmunisation is an important complication resulting from chronic blood transfusions. Studies investigating the effects of alloimmunisation on the lymphocyte counts of patients with SCD have reported significant changes [24,47,53,54,55,56]. One such study demonstrated a significant decline in regulatory CD4+ T lymphocytes and an increase in regulatory CD8+ T lymphocytes [57], suggesting that these patients may be at risk of developing autoimmunity.

Further investigations of the B cell lineage have demonstrated that they are also functionally abnormal. Abnormalities include decreased antigen-specific B cell proliferation and IgM secretion. According to Ochocinski et al. [29], defects in B cell lymphocyte function in children affect the production of natural anti-polysaccharide antibodies, making children with SCD more susceptible to infection and disease. In a study where the effects of haemolysis were examined on human B- cell responses and alloimmunisation risk in SCD patients, two pathways were tested and included the STAT3 and HO-1 pathways. This study reported that heme inhibits human B-cell differentiation by modulating HO-1 enzymatic activity through blockage of the DOCK8/STAT3 signalling pathway. It was also reported that the B cells from SCD alloimmunised patients are resistant to heme inhibitors as well as reversed resistance with quinine [58].

## 3. Autoimmunity in Sickle Cell Disease

**Figure 1:**
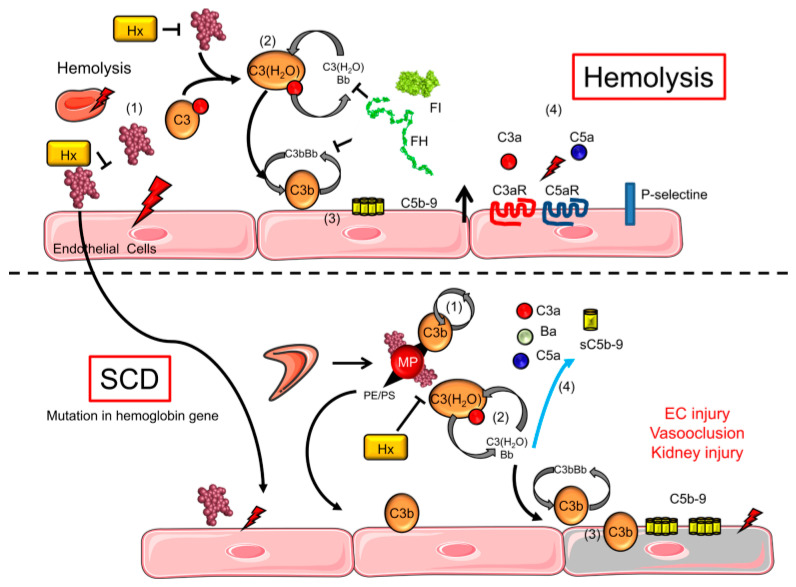
Complement activation pathway and haemolysis in SCD patients [61].

Autoimmunity is a disorder in which the immune response is directed against its normal body constituents such as cells and tissues. Any disease resulting from this dysregulated immune response is termed an autoimmune disease and can be caused by antibodies produced by B lymphocytes or the action of T lymphocytes directed against normal tissue. Patients with SCD often exhibit aberrant activation of the alternate complement pathway, which can lead to higher risks of infection and is thought to predispose patients to autoimmune disease (AID) [59]. The mechanisms resulting in the abnormal activation of complement include the association of complement with cell-free heme and haemolysis-derived molecules. P-selectin together with C5a (a complement regulatory protein), the hypercoagulant environment, the continual damage and correction of the red cell membrane structure as well as the vicious cycle of ischemia perfusion (IP), all result in complement activation through diverse pathways [60,61,62]. Circulating microvesicles (MVs) of damaged sickled RBC during oxygenation–deoxygenation sickling cycles have been identified as having the capability to trigger the complement cascade [61,63]; see Figure 1.

In healthy individuals, the prevalence of antinuclear antibodies varies with incidences of between 12 and 30% [64], while in Africa, the prevalence is higher and ranges from 7 to 39% [65]. 

In patients with SCD, high autoantibody titres have been observed even in the absence of clinical autoimmunity [66,67]. The mechanisms leading to the formation of these autoantibodies are unclear but may involve impaired splenic function. The spleen has several purposes which include the removal of old and damaged red cells as well as the regulation of the immune response. The importance of the spleen in preventing the development of autoimmune processes has been demonstrated in studies which have documented high autoantibody titres after splenectomy [68]. Moreover, chronic inflammation [28] and alloimmunisation by multiple transfusions have also been implicated in the development of autoantibodies, but these have not been fully confirmed in clinical studies [27,66,67]). The coexistence of SCD and autoimmune disease is difficult to treat as those receiving steroids, which are used to control the inflammatory response, experience repeated VOC [69,70,71]. In confirmation of this, Bernini et al. [72] reported on four cases of SCD who received increased doses of corticosteroids during pain crises and chest pain syndrome. Although the treatment shortened the period of complication, the high dosage resulted in severe VOC episodes and haemorrhagic stroke. Likewise, recurrent blood transfusions or exchange transfusions, although assisting in the prevention of VOC, lead to increased risks of antigenic alloimmunisation [73]. Biological therapies such as anti-TNF, hydroxyurea and haemopoietic stem cell transplantation may be better therapeutic choices [74].

## 4. The Role of Oxidative Stress in the Pathogenesis of Sickle Cell Anaemia

**Figure 2:**
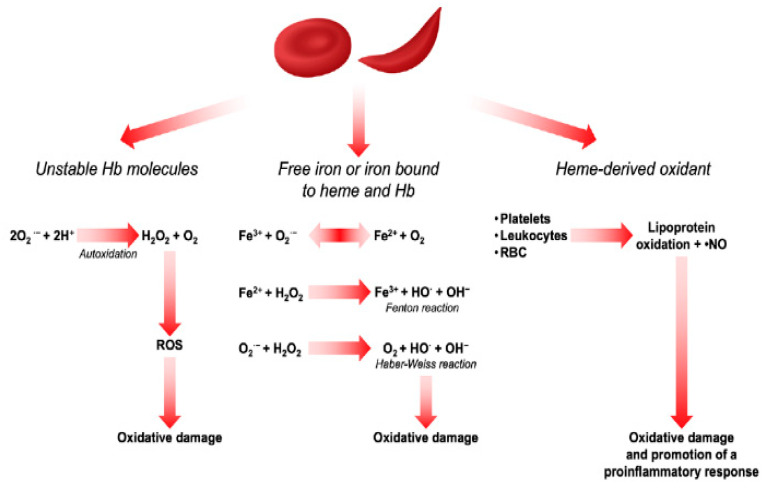
Sources of ROS in RBCs (adapted from [80]).

Oxidative stress is an important contributor to the pathogenesis of sickle cell anaemia (SCD) and associated complications such as sickling, vaso-occlusion, and ischemia–reperfusion injury [2,27,36,75,76]. Oxidative stress occurs due to an imbalance between the production of reactive oxygen species (ROS) and reactive nitrogen species (RNS) and the ability of antioxidant agents, including enzymes such as superoxide dismutases, catalase, and glutathione peroxidase, to neutralise them [28,36,77]. Patients with SCD are frequently exposed to oxidative stress, and studies have found higher levels of ROS in the RBCs of SCD patients compared to healthy controls. The concentration of the reactive intermediates generated from the oxidative reactions has often been used as markers of disease severity [28,78], and the mechanisms leading to oxidative stress in SCD patients’ red blood cells (RBCs) are well established. Some of these include haemoglobin (Hb) autoxidation. When SaQ1234567890 */haemoglobin is released into the bloodstream as a result of haemolysis, superoxide (O_2_) produced, which can dismutate into hydrogen peroxide (H_2_O_2_) and serve as a starting point for additional oxidative reactions [9,79]. Apart from haemoglobin (Hb) oxidation, other factors enhancing ROS production include ischemia–reperfusion injury caused by oxygen deprivation [12], which has been reported to promote the activation of pro-inflammatory mediators such as xanthine oxidase, NADPH oxidase, nitric oxide synthase, and lipoxygenase [2,28]. Another factor contributing to the excessive ROS in SCD patients is the release of iron and heme from unstable HbS, which may catalyse the Fenton reaction. Iron (II) will react with hydrogen peroxide ions leading to the formation of ion (III) and hydroxyl radical [12]. Figure 2 illustrates each of the above mechanisms. 

To counteract radicals, the body produces antioxidants [38,81,82]. These include non-enzymatic antioxidants, such as microelements carotenoids and ascorbic acid [83], and enzymatic antioxidants including dismutase, catalase, glutathione peroxidase and heme oxygenase-1 [2,28]. However, due to the high levels of oxidative stress in SCD patients, antioxidants are overwhelmed by the continual source of ROS. Some unneutralised ROS have been reported to oxidise membrane lipids, proteins, and DNA, causing cell death and organ damage [84]. This damage leads to further ROS production, thereby aggravating the disease. The oxidative damage to lipids known as lipid peroxidation happens when membrane phospholipids are exposed to a hydroxyl radical (HO^•^) and hydroperoxyl (HO^•^ _2_), which have been reported as the two most prevalent ROS affecting lipids [85,86]. During lipid peroxidation, highly toxic molecule end products, including malondialdehyde (MDA) and 4-hydroxy-2-nonenal (HNE), can easily interact with proteins and DNA, causing damage [2,83]. Malondialdehyde (MDA) is an important marker for evaluating oxidative stress in patients with SCD [28]. A study in Cameroon observed an increase in MDA in SCD patients compared to healthy individuals [87]. Similarly, in Ghana, Antwi-Boasiako et al. [36] reported that MDA levels were significantly higher in SCD patients with VOC, which was followed by patients in steady-state. F_2_-isoprostanes, as a marker of oxidative stress, has been reported to be higher in sickle cell patients compared to healthy controls [88,89]. Nader et al. 2020 [90] assessed the contributions of NO and oxidative stress on eryptosis (apoptosis of red cells) and the release of RBC microparticles (RBC-MPs) on vascular dysfunction. It was reported that oxidative stress initiates eryptosis, and the release of MPs, generated during this process, may be significant in microvascular dysfunction. RBC-MPs could be harmful to the microcirculation’s endothelial cells by activating Toll-like receptor-4 (TLR4) and promoting the expression of adhesion molecules as well as the release of cytokines which in turn fuels vascular dysfunction. Although this investigation offers fresh insight into the underlying processes of vascular dysfunction in SCD, more research is required. New therapeutic targets that aim to prevent eryptosis and/or TLR4 activation are suggested [90].

Oxidative stress in SCD is associated with worsening symptoms, including accelerated haemolysis [91], endothelial damage [85], decreased NO bioavailability [2], and hypercoagulability [92]. Oxidative stress is inevitable in a patient with SCD; however, antioxidant therapeutic strategies, including the use of L-glutamine, N-acetylcysteine, and manganese porphyrins, have the potential to reduce the detrimental effects [75].

## 5. The Role of Inflammation in the Pathogenesis of Sickle Cell Anaemia

Inflammation is the body’s natural response to toxic chemicals, infection, and injury. Although it is difficult to determine the exact events that trigger the chronic inflammatory state in sickle cell disease (SCD), some mechanisms have been reported [31,45]. The sources of inflammation in SCD include red cell alterations, haemolysis, vaso-occlusive processes, ischemia–reperfusion injury, infections, release of histamine, oxidative stress, thrombin generation and activation of complement [31,89]. Many reported complications such as acute chest syndrome, stroke, leg ulcers, nephropathy, and pulmonary hypertension have been linked to inflammatory processes [93].

Haemolysis is the major inflammatory trigger affecting the bioavailability and function of anti-inflammatory molecules such as nitric oxide (NO) and heme oxygenase 1 (HO-1) [92,94,95]. Heme oxygenase 1 (HO-1) is an enzyme with numerous anti-inflammatory properties, including the breakdown of heme and the generation and release of reaction products, including carbon monoxide, ferrous ions, and biliverdin [96,97,98,99,100]. Continuous haemolysis leads to the overproduction of heme, which in turn accelerates the HO reaction, causing an excessive accumulation of reaction products, and if not sufficiently sequestered, it will have serious consequences [96]. During haemolysis, free haemoglobin and heme destroy nitric oxide (NO) produced by endothelial nitric oxide synthase. Nitric oxide functions in preventing endothelial activation as well as controlling leukocyte activation and emigration from blood vessels to tissue [2]. Researchers have demonstrated that free haemoglobin in the plasma destroys NO 1000-fold faster than haemoglobin encapsulated within the red blood cells [9]. 

Neutrophils are one of the first cells to respond to infections. Their movement to the site of injury is triggered by Pathogen-Associated Molecular Patterns (PAMPs) from microbes or Damage-Associated Molecular Patterns (DAMPs) derived from damaged host cells. Activated neutrophils release ROS, proteases, myeloperoxidase, defensins, cathepsin G, and elastase to combat foreign organisms at the site of infection [101]. These enzymatic proteins are all involved in inflammatory processes [102] and when cell adhesion takes place, chemokines and cytokines are produced which go on to stimulate dendritic cells resulting in the presentation of antigens to memory CD4^+^ T cells as well as to naïve CD8+ T cells. This leads to activation of the adaptive immune response [103]. 

ROS, produced by activated neutrophils, hinders the function of effector NK cells, while GM-CSF cytokines and IFN-γ produced by NK cells prolong the survival of neutrophils in an in vitro system [104]. 

Due to the prominent role neutrophils play in the inflammatory response, it has been hypothesised that they could be important in the response to plasma methaemoglobin produced during haemolysis. This was investigated, and the results showed that methaemoglobin is an endogenous DAMP ligand for TLR2 and that neutrophils actively respond to the (metHb + LTA) induced production of ROS. Interestingly, it was also observed that this response diminishes in the presence of other white cells, indicating that cells of the immune system communicate with each other to modulate cellular responses during a haemolytic reaction [105]

Vaso-occlusive crises (VOCs) occur when sickled red blood cells obstruct blood flow to the tissues [24,106,107]. This, in turn, triggers an inflammatory reaction as the body attempts to correct the condition. In SCD, vaso-occlusive processes generate ischemia–reperfusion injury, known as tissue damage, which is caused by a disruption in blood supply [2,107,108]. Ischemia–reperfusion damage increases oxidant generation and leukocyte adhesion, contributing to chronic inflammation.

Transforming growth factor (TGF-), interleukin-17 (IL-17), tumour necrosis factor (TNF-), IL-6, and IL-8 are all significantly elevated in steady-state SCD patients when compared to controls. These studies all confirm the chronic inflammatory environment present in patients with SCD [40,108].

## 6. The Relationship and Interdependence between Inflammation and Oxidative Stress in SCD

Comprehensive studies have demonstrated that oxidative stress and inflammation are closely linked [81,86,109]. Both mechanisms occur concurrently in many pathological conditions, including sickle cell disease (SCD), resulting in a vicious cycle that aggravates the disease [9,110]. Several factors contribute to the overproduction of ROS in SCD, which, if not immediately sequestered, can create a chain reaction that results in chronic inflammation [12,81]. Oxidative stress can activate a wide range of transcription factors and receptors, such as nuclear factor-κB (NF-κB) and activator protein-1 (AP-1) [80,81], which control the expression of a wide variety of genes, including those responsible for producing pro-inflammatory and anti-inflammatory cytokines [80,111].

Pattern recognition receptor Toll receptor 4 (TLR4) triggers the innate and adaptive immune response by promoting the secretion of pro-inflammatory cytokines like TNF, IL-1, IL-6, and IL-12. This process can be activated by oxidative stress and thus leads to inflammation [76,112]. Cell-free haemoglobin, derived from sickle RBCs, contributes to vascular dysfunction by promoting inflammation via the activation of TLR4 [9,12]. In addition to the direct activation of transcription factors and receptors, Damage-Associated Molecular Patterns (DAMPs) released during haemolysis cause damage to some biomolecules and promote inflammation through the NF-*κ*B pathway [113]. Hydrogen peroxide, for example, can react with nitric oxide (NO) to form peroxynitrite, which is a highly reactive oxidising and nitrating agent capable of damaging lipids, DNA, and protein. These reactions promote cellular necrosis and apoptosis [31]. 

**Figure 3:**
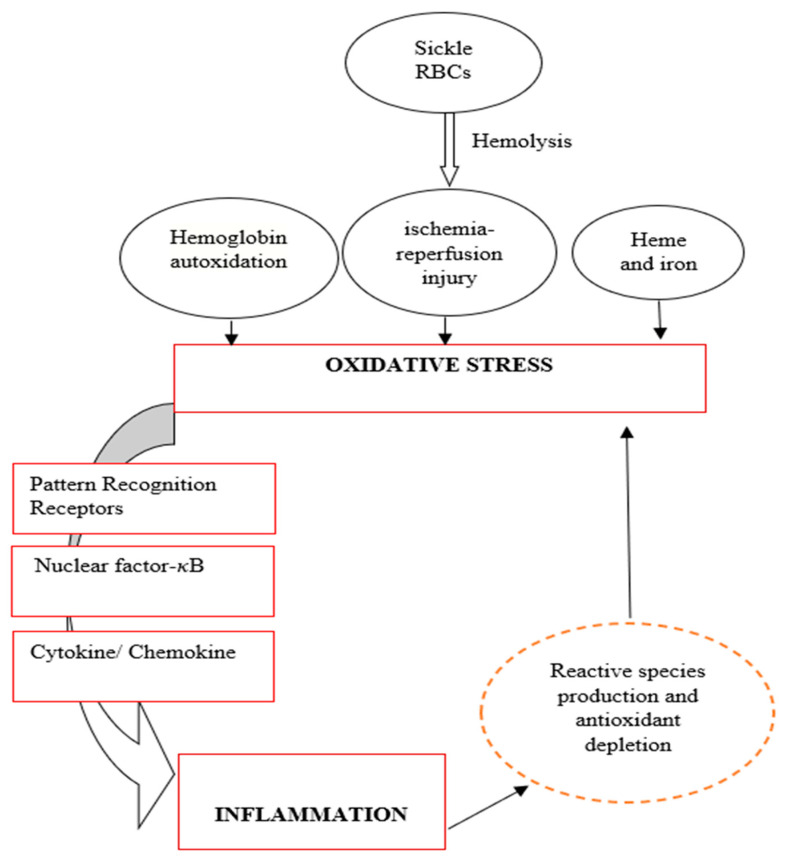
Relationship between oxidative stress and inflammation in the pathogenesis of SCD.

On the other hand, chronic inflammation can induce oxidative stress through the continuous activation of immune cells [76,81,114]. At the site of injury, immune cells such as phagocytic and non-phagocytic cells release reactive oxygen species, chemical mediators, and enzymes contributing to higher oxidative stress [115,116,117]. Nonphagocytic cells have been reported to generate reactive species in response to pro-inflammatory cytokines, leading to an imbalance between pro-inflammatory and anti-inflammatory cytokines, leading to oxidative stress [27,86]. Figure 3 depicts the relationship between oxidative stress and inflammation in SCD. If oxidative stress appears as the primary abnormality, it will stimulate inflammation, which will further induce oxidative stress and vice versa. 

Due to the interaction between oxidative stress and inflammation, some researchers have discovered that using antioxidants to treat only oxidative stress may not always be successful [2,15,75]. Inflammation and oxidative stress work together to amplify each other and cause progressive damage once the process begins. Finding antioxidants that can simultaneously prevent oxidative and inflammatory pathways has proven to be difficult. Hence, a comprehensive understanding of these pathological events could contribute to developing novel therapeutics.

## 7. Inflammation and Blood Transfusion in SCD

Numerous therapies have been used successfully in treating sickle cell disease such as hydroxyurea, gene therapy, and stem cell transplantation; however, blood transfusion remains the most effective therapy [93] despite the disadvantages and risks. These risks include alloimmunisation, blood-borne diseases and iron overload. The build-up of alloantibodies [118] is most likely due to incompatibility in antigenicity between donors and recipients and may lead to delayed transfusion reactions [56].

## 8. The Cause of High Alloimmunisation in SCD Patients

Alloimmunisation is prevalent in SCD patients and increases with the number of blood transfusions, the age at the first transfusion, genetics and sex [54]. Thompson et al. [54] linked high alloimmunisation in SCD patients to the presence of chronic inflammatory disorders which triggers the development of autoantibodies and alloimmunisation. A low expression of CD64 (FcyR1) in classical and intermediate monocytes and the inflammatory milieu found in SCD patients have also been shown to contribute to their high alloimmunisation [119]. Another reason for the development of antibodies is the mismatch between the donor and the recipient, which becomes important when choosing donors. Reports from studies in Uganda, Burkina Faso, and Egypt showed that alloimmunisation is lower possibly because both donor and recipient belong to the same ethnic group [56,93]. This hypothesis was supported by a study conducted in Cape Town which reported an increase in alloimmunisation attributed to donors and recipients being from different ethnicity [54]. Other mechanisms implicated in the development of alloantibodies could be iron overload and pregnancy.

Pretransfusion antibody screening typically includes Rhesus and ABO grouping. However, other blood group systems, including Kell, Kidd, Duffy, Lewis, Lutheran, P, and MNS], often regarded as minority or weak blood groups, have been linked to alloimmunisation or antibody formation [120,121]. Considering this, it has become imperative that routine blood grouping should include other blood group antigens for effective, complete, and accurate pretransfusion screening. This is crucial in the case of sickle cell disease patients who require multiple blood transfusions [36,122]. Patients with sickle cell disease are more at risk of developing alloimmunisation, which makes cross-matching and suitable blood for transfusions problematic when the issues of minor antigens are not considered during the transfusion [58,119]. According to Boateng et al. [123], the frequency of alloantibody development in patients with SCD is as high as 76% compared to the general population [124]. The frequency of red blood cell alloimmunisation in SCD patients may not be the same in every part of the world due to blood transfusion rates, ethnic mismatch, and the age of the initial transfusion [20,125,126].

## 9. Other Treatment Options 

The US Food and Drug Administration has approved HU, L-glutamine, crizanlizumab, and voxelotor to reduce the acute complications of SCD [24,31]. Hydroxyurea is the most commonly used of these, while other drugs, including L-glutamine and crizanlizumab, have not been widely adopted despite European approval [127]. Additionally, although HU is effective in reducing acute complications improving quality of life, and organ function and prolonging survival, it also remains underutilised primarily due to inexperience and unfounded safety concerns [128]. Although the mechanism of action of HU is still unclear, previous studies have shown that after treatment, nitric oxide (NO) production is improved and the concentration of foetal haemoglobin a(HbF) in erythrocytes is enhanced, thereby preventing HbS polymerisation [11,129]. Several studies have investigated the effectiveness and safety of these drugs in reducing the frequency VOC and inflammation in SCD patients [130,131,132,133] and have reported that voxelotor increases haemoglobin levels, does not impair oxygen delivery, reduces hospitalisation for VOC and decreases sickle red blood cell levels. It has been reported that L-glutamine and crizanlizumab reduce VOC episodes and prolong the time between the first and second pain crises. In this study, it was reported that inflammatory molecules were reduced with HU therapy in children with sickle cell SCD [40]. Others have supported this and have observed that patients receiving HU had lower interleukin IL-6 levels [134,135]. In contradiction, however, others have reported elevated levels of interleukin (IL)-6 compared to untreated patients [82,106,136,137,138].

## 10. Conclusions

Sickle cell disease is a haemolytic anaemia in which the red cells have a shortened life span. Patients suffer frequent haemolytic episodes, leading to the release of heme and haemoglobin into the circulation, which reduces the availability of anti-inflammatory molecules. This initiates a series of events with the activation of immune cells and oxidative stress, which is often amplified by multiple transfusions and the development of autoantibodies. The frequent recurrence of these processes leads to the development of a pro-inflammatory and pro-coagulant environment which predisposes patients to the development of complications such as stroke. 

This manuscript has attempted to review the current knowledge and understanding of the unique mechanisms in SCD which led to a dysfunctional immune response. Although much research has been conducted, the pathways leading to the observed inflammatory state remain unclear and require further investigation. Further clarity on these mechanisms may result in the development of therapies which could prevent the development of complications observed in patients with SCD. Also unravelling the complex relationship between the immune response and the pathogenesis of sickle cell anaemia could result in the development of novel therapies which would target inflammatory pathways.
